# The Hidden Effects of Dairy Farming on Public and Environmental Health in the Netherlands, India, Ethiopia, and Uganda, Considering the Use of Antibiotics and Other Agro-chemicals

**DOI:** 10.3389/fpubh.2016.00012

**Published:** 2016-02-24

**Authors:** Maria J. Groot, Katrien E. van’t Hooft

**Affiliations:** ^1^RIKILT, Wageningen University, Wageningen, Netherlands; ^2^Dutch Farm Experience, Maarsbergen, Netherlands

**Keywords:** dairy farming, antibiotic resistance, pesticides, milk quality, herbal medicine, Holsteinization

## Abstract

The current and expected growth of the world’s population warrants an increased production of high-quality animal protein. Dairy farming is regarded as one of the important ways of satisfying this need to meet the growing demand for milk, especially in developing countries. The focus on crossbreeding and increasing the productivity of dairy cattle has, besides enhanced milk production, also resulted in an increased use of agro-­chemicals, mainly antibiotics and anti-parasite drugs. The residues of these agro-chemicals, if not managed properly, could leak into the environment, affecting natural processes, biodiversity, and soil life. Public health can also be affected due to residues in milk and meat, especially in countries with insufficient food quality controls. These processes contribute to the growing global threat to human and animal health posed by multi-resistant microbes. This article discusses the differences and similarities of dairy farming, and the effect on public and environmental health, between the Netherlands, India, Ethiopia, and Uganda, emphasizing the strategies that have been developed during the E-Motive exchange project to reduce the use of antibiotics and other chemicals in dairy farming. Proposed solutions include raising consciousness about the risk of antibiotics and their effect on food quality, and implementing the Natural Livestock Farming five-layer approach for reducing the use of antibiotics and other chemicals. This approach is based on improving animal and farm management, revitalizing ethno veterinary knowledge and the use of medicinal plants, genetic improvement through strategic use of local breeds, establishing quality control systems in the dairy chain, and extra payment to farmers for residue-free milk.

## Introduction

The current and expected growth of the world’s population warrants increased production of high-quality animal protein. Dairy farming is regarded as one of the important ways of satisfying this need, especially in developing countries (1). Therefore, dairy development programs have been started aiming at increasing animal productivity by crossbreeding with high yielding breeds or their introduction, especially of the Holstein-Friesian (HF) cattle. Through continued crossbreeding, they gradually replace local cattle breeds that produce less milk but which are better adapted to local environment. Moreover, local breeds combine a variety of purposes, including production of milk, meat, traction, and manure to fertilize the fields. This common strategy of replacing local cattle breeds with HF cattle is also known as the “Holsteinization” of dairy farming (2).

The focus on crossbreeding and increasing the productivity of dairy cattle also results in an increased use of agro-chemicals, mainly antibiotics and anti-parasitic drugs. The residues of these agro-chemicals come into the environment, affecting natural processes and soil life (3). Public health is also affected due to residues in milk and meat, especially in countries with insufficient food quality controls (4). There is also evidence of a global rise in multi-resistant microbes due to the extensive and inappropriate use of antibiotics, and the use of antibiotics in animal production, including dairy farming, is a contributing factor (5–8). The use of antibiotics in animal production is a global issue and is not confined to developing countries.

Since their introduction in the 1940s, antibiotics have been very important in modern healthcare. Now, however, once-treatable infections are becoming difficult to cure, which increases costs, morbidity, and mortality in both humans and animals. Antibiotic resistance is a direct result of antibiotic use: the greater the volume of antibiotics used, the greater the risk of antibiotic-resistant populations emerging. Some classes of antibiotics, such as carbapenems and cephalosporins, are used as last resort for infections. Cephalosporins are one of most widely used drug classes worldwide, and the latest developed drugs are numbered third- and fourth-generation cephalosporins. These drugs are used as last resort for serious infections in humans due to food-borne pathogens *Salmonella* and *Shigella* (5). It is of the utmost importance to preserve the usefulness of antimicrobials in treatment of human disease. One of the means of doing this is to exclude them from veterinary use (8). According to the Dutch guidelines for veterinarians, the use of last resort antibiotics (carbapenems, glycopeptides, oxazolidones, daptomycine, mupirocine, and tigecycline) for animals is forbidden (9).

Antibiotic resistance can be developed by bacteria using different genetic strategies, such as producing destructive enzymes to neutralize antibiotics; mutation, so that drugs cannot recognize their targets; pumping antibiotics out of the cell (efflux); creating a “biofilm” so antibiotics cannot reach them; and creating bypasses so bacteria can function without the enzymes targeted by antibiotics (10).

The growing challenge of antimicrobial resistance is mainly due to the high use of antibiotics in human health care and their availability, also in developed countries (5–8). Other contributing factors include the high use of antibiotics in animal production and multi-resistant strains of microbes in animal farming across the globe. A recent scientific review in the UK indicates that, if no solutions are found, multi-resistant microbes will result in a global crisis with more human deaths in 2050 than cancer today – the majority in the poorer regions of Africa and Asia (11).

The intensification of animal production has also had a considerable impact on the environment. The current loss of biodiversity is devastating. One of the major causes of this phenomenon is habitat loss and modification as a result of intensified agricultural practices (12).

In 2014 the Dutch Association of Phytotherapy (NVF) and Dutch Farm Experience (DFE), a company that links international organizations to sustainable Dutch dairy farming, initiated the E-Motive exchange project between the Netherlands and India to reduce the use of antibiotic in dairy farming. Funded by Oxfam-Novib and the province of Overijssel, a group of farmers, veterinarians, and researchers from both countries visited each other over a period of 2 weeks. This resulted in increased awareness of the problem as well as a joint search for strategies in animal management, herbal medicines, and breeding that would reduce the use of antibiotics. In this process, Indian expertise on medicinal plants and ethno veterinary medicine is being combined with Dutch expertise on farm management and milk quality control. Due to the promising outcome of this Dutch–Indian exchange, the project was extended to 2015, and included two African countries, Ethiopia and Uganda, with special emphasis on cattle breeding.

This article discusses the differences and similarities in dairy farming in the Netherlands, India, Ethiopia, and Uganda and its effect on public and environmental health. It emphasizes the strategies that have been developed during the E-Motive exchange project to reduce the use of antibiotics and other chemicals in dairy farming. The impact of antibiotic use on human health through antibiotic use, the growing problem of antibiotic resistance, and the lack of adequate control systems are discussed as well as the effects of dairy farming on the environment. These include agro-chemical pollution and subsequent loss of biodiversity, changes in pasture management, and the loss of genetic diversity and local breeds. A five-layered strategy based on the experiences in the four countries is proposed to stimulate a more sustainable and healthier approach to dairy farming and to help reduce the use of antibiotics and improve milk quality.

## Dairy in Netherlands, India, Ethiopia, and Uganda

In the Netherlands, the focus on specialized milk production since the 1960s has led to cows that produce extremely high milk yields. This requires high inputs of maize and concentrated feed, as well as fodder from high yielding monoculture grasslands. The cattle manure, combined with urine obtained from the commonly used housing systems, is injected into the soil. This way of fertilization prevents the excessive emission of ammonia into the air, but has negative effects on soil life and soil fertility (13). The pastures with high yielding grass monocultures, especially English Ray grass, have resulted in the loss of meadow birds, insects, and other soil life. Moreover, this high-input, high-output dairy management system has led to shortening the life span of the animals due to a high incidence of udder infection (mastitis), claw problems, and infertility (14–16). Intensification has led to high antibiotic use in livestock production in general; in dairy farming especially to the need to cure claw problems and calf scour, and to treat and prevent udder infections. In the Netherlands, strict (milk) quality controls on antibiotics and other chemicals are in place and this guarantees good quality dairy products. Over the past decade, awareness about the risk of multi-resistant microbes has grown, and since 2012 government regulations now severely limit the use of antibiotics in livestock production systems. Antibiotic usage and resistance in both humans and animals is being monitored (17, 18).

**Figure 1:**
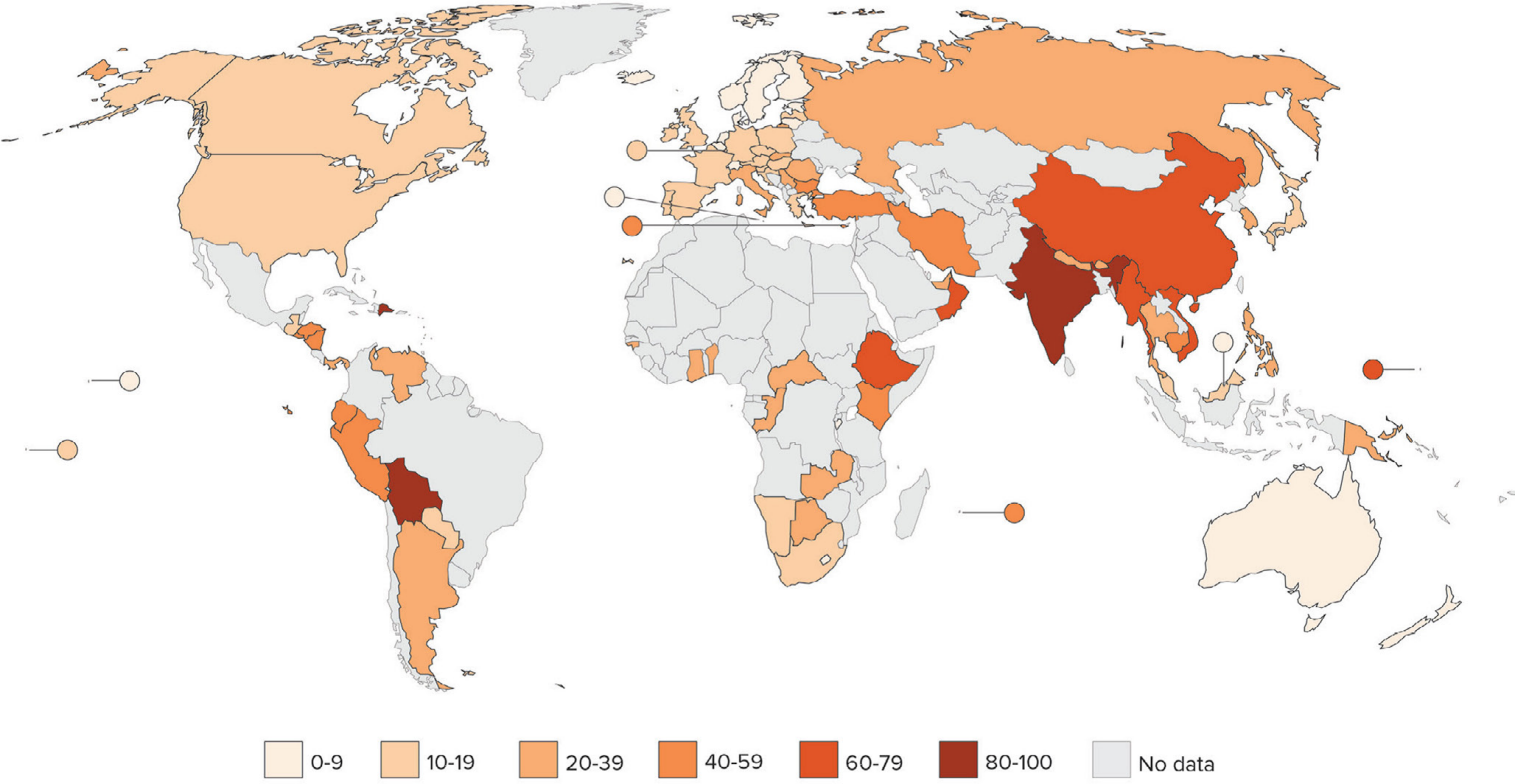
Percentage of extended-spectrum beta-lactamase producing Escherichia coli*, by country (most recent year, 2011–2014). Source: CDDEP 2015, WHO 2014 and PAHO, forthcoming.

In India, the National Dairy Development Program based on continued crossbreeding local breeds with HF semen has been taking place since the 1980s. India is now the worlds’ largest milk producer with the milk coming primarily from smallholder mixed farms with one to three cows. Meanwhile disease problems, especially udder infection, indigestion, and infertility have resulted in the increased use of antibiotics and hormones. The importance of dairy cattle for rural families and the severity of cattle diseases, combined with the uncontrolled availability of antibiotics, makes antibiotics the drug of first choice, including the use of third- and fourth-generation antibiotics that are to be safe-guarded for human use. India is now among the countries with the highest levels of resistant bacteria in humans (5), see Figure 1.

A recent study indicated that, between 2010 and 2030, the estimated global consumption of antimicrobials in livestock will increase by 67% (19), although this increase is mostly attributed to pigs and especially poultry. Meanwhile, there is no systematic control of residues in the milk and milk quality is affected due to chemical residues (4, 20).

The dairy improvement program in Ethiopia was initiated in 2000 to deal with the increased need for animal protein of the explosively growing population. Most of the HF and crossbred dairy cattle introduced are kept indoors by smallholder producers. The animals are fed with roughage from grazing, hay and crop production, and supplements, including wheat bran and concentrates from oil processing plants. In the highlands, the manure of these animals is dried and sold for combustion; while in some areas, it could also be used for composting. Due to the combination of a lack of breeding strategy and mismanagement, many of the crossbred cows suffer from udder infection (mastitis), claw problems, and infertility (21–23). The Ethiopian Livestock Master Plan (LMP) has laid out a road map that details a strategic breeding program that delineates agro-ecological settings where local cattle selection and breeding is to be carried out and areas suitable for crossbreeding, thus, addressing the danger of indiscriminate crossbreeding (24). There is documentation on ethno veterinary practices in some regions of Ethiopia, which could be evaluated and implemented elsewhere (25).

In recent years, in Uganda, the local Ankole cow has been increasingly crossbred with HFs to boost milk production. Generally, these crossbred cattle graze outside, and besides relatively high-quality feed they also require intensive tick protection to survive. If they are not protected, the animals will suffer from tick-borne diseases, such as East Coast fever. Therefore, all cattle are sprayed up to two-times a week with acaricides against ticks. Local farmers have seen that this has had a serious effect on the habitat of bees and butterflies, as well as insect eating birds, such as ox peckers and white herons. Moreover, soil life is affected, as is the health of the people exposed to these pesticides. Both acaricides and antibiotics are generally used without any form of control (26, 27).

## Current Situation

Table S1 in Supplementary Material provides an overview of the current situation in the countries involved.

Despite the differences in milk production, environmental problems occur in all countries (23, 26, 28, 29). Public health also continues to be of vital concern especially in those countries with inadequate food (milk and meat) quality controls.

## Oxfam Exchange Project: Some Outcomes

The aim of the exchange project was to work toward developing long-term engagement to reduce antibiotic use in dairy production by combining the expertise from various countries, especially knowledge on medicinal plants [also known as ethno veterinary practices (EVP)], animal management, breeding and milk quality control. Generally speaking, the exchange has inspired the participating veterinarians, animal scientists, farmers, and researchers to the extent that various initiatives to reduce the use of antibiotics in dairy have been taken in all the countries.

During the first year of the project, it became clear that India has extensive knowledge about medicinal plants, which when compared to other countries is relatively well-accepted by farmers and veterinarians (30–40). For example, during a severe foot-and-mouth disease (FMD) outbreak in 2013, a herbal remedy was ­designated the first choice protocol by the state government of Kerala. The Trans-Disciplinary University (TDU) in Bangalore and the Tamil Nadu veterinary Science University (TANUVAS) in Chennai have documented and validated effective herbal remedies for the 15 main clinical conditions in dairy farming, including mastitis and diarrhea (33, 41, 42), as has Anthra (43), and these institutes are providing training on the use of medicinal plants to farmers and veterinarians in three states in South India: Karnataka, Kerala, and Tamil Nadu. Between 2013 and 2015, 240 milk samples from these three states were analyzed for residues of antibiotics by TDU and TANUVAS. This study showed that the use of Ayurvedic remedies to treat animal diseases reduced the number of antibiotic-positive milk samples by between 18 and 49% in the three states (44).

The experience with medicinal plants was surprisingly well received by the Dutch delegation, and afterwards a trial was organized in the Netherlands to validate the use of one herbal recipe for high cell count in dairy cows. A high cell count in the milk is an indication of subclinical mastitis, a situation that often occurs in high production cows and leads to reduced milk production (45). Though this remedy was not used for clinical mastitis as it is used in India, it did give a significant reduction in the cell count of treated cows when compared to the control group. This experiment has given a boost toward a wider acceptance of herbal products in Dutch dairy farming.

An important outcome in India is the initiative of two major dairy companies (Karnataka Milk Federation and MILMA) to improve milk quality by starting pilot “organic” milk production projects based on a five-leveled approach that combines Dutch and Indian expertise: (1) improved animal and farm management; (2) strategic use of local cattle breeds (3) use of medicinal plants; (4) milk quality control system at village level; and (5) extra payment to farmers for residue-free milk.

The results of these experiences have also been captured in two films [*Africa’s Milk Dilemma* (46) (about Uganda), and *Green antibiotics* (47)]. Other forms of communication to raise awareness of the risks of modernization (i.e., Holsteinization) of dairy farming in relation to environmental and human health are being developed. These publications are aimed at veterinarians, farmers, researchers, and policymakers in the four countries involved in the exchange. The threat of multi-resistant microbes and antimicrobial resistant (AMR) infections is still largely unaddressed within the dairy sector in India, Ethiopia and Uganda, while the use of antibiotics and other agro-chemicals is rampant and largely uncontrolled (48, 49).

## Impact on Public Health

The primary aim of the exchange project between India and the Netherlands was to reduce the use of antibiotics to improve animal management and to revive the use of ethno veterinary medicine as a means of reducing the need for antibiotics. But when the African countries became involved, the problem appeared to be more complex and a more holistic view on dairy farming and the development of more intensive systems was required. In Africa, it appeared that besides uncontrolled antibiotic use, the inappropriate and excessive use of acaricides is having a devastating impact on the environment. Another factor that contributes to the heavy use of agro-chemicals is a breeding strategy in which local breeds are gradually replaced by breeds with a higher production capacity, indiscriminate crossbreeding resulting in animals that are less suited to the local environment and that need extra care and medicines. In the following sections, the experiences of the exchange project and data from relevant literature are provided to give a more in-depth overview of the effects of dairy farming on public and environmental health.

### Growing Resistance to Antibiotics for Human Treatment

The antimicrobial multi-resistant infections in animals that threaten human health are zoonotic pathogens transmitted through food, especially *Salmonella* and *Campylobacter*, and through the environment (e.g., ESBL coli in water). Livestock-associated methicillin-resistant *Staphylococcus aureus* (LA MRSA) and extended-spectrum beta-lactamase *E. coli* (ESBL *E. coli*) are also emerging problems throughout the world. Grace (6) states that there is a lack of accurate information on antibiotic use in developing countries, but agricultural use is thought to exceed medical use. Most use is expected in intensive production systems (pigs, chicken) – sectors that are increasing rapidly due to the growing demand for animal protein. The few reviews that investigate the occurrence of AMR in zoonotic disease show figures from 37 up to 100% for ampicillin and 0–43% for newer antibiotics, such as ciprofloxacin (5, 6). Factors influencing the development of AMR are the uncontrolled sale of antibiotics, a lack of awareness and concern, the lack of information, fake and substandard drugs, poor integration between human and animal health systems, and the lack of alternatives for antibiotics.

### Milk Quality

In most countries, milk quality is assessed according to somatic cell counts, solids (fat and protein) content, and the presence of unwanted residues. There was a lack of control mechanism as well as a lack of capacity to detect chemical residues in milk in three of the four countries participating in the exchange (India, Ethiopia, and Uganda), which severely affects milk quality. In fact, we found many cases in which dairy farmers keep local breed cattle for home consumption, while selling the milk from crossbreed cattle on the market. They are aware of the risks of residues, but are not able to translate that into residue-free milk for the market and consumers. During the exchange, we tested milk and milk products from milk collection points as well as supermarkets, and found worrying levels of residues. Besides the threat to human health, there is also a threat to the local dairy market: consumers may decide to go only for imported (quality controlled) milk. Like the case of Chinese dairy production, where consumers have moved away from baby milk powder produced in China that might be contaminated with melamine and are prepared to pay exorbitant prices for milk powder from Europe (50, 51). However, not all imported products meet the quality criteria, as is the case in China where milk powder from New Zealand was banned due to a Botulism scare (52).

## Impact on the Environment

### Leakage of Antibiotics into the Environment

The large-scale use of antibiotics affects the bacteria in the soil and water (53). For example, the antibiotic Sulfamethoxazole in concentrations previously found in aquatic environments (approximately 1 μg/L) delays the start of cell growth, limits denitrification (a critical component of global nitrogen cycles) and alters bacterial community composition. Adverse effects on soil bacteria in tests conducted at higher antibiotic levels (250 μg/L or greater, sub-therapeutic levels) were even more pronounced. Denitrification is the microbial facilitated process that converts nitrate to nitrogen gas, and also is of major importance for soil fertility as well as in the natural assimilation of nitrate pollution (53).

### Loss of Biodiversity

Thomassen (54) investigated the environmental impact of dairy cattle production systems using assessment by indicators derived from input–output accounting (IOA), Ecological Foot Print analysis (EFP), and life-cycle assessment (LCA). Different ways of milk production exist, such as milk produced in a conventional or organic dairy cattle production system. Thomassen showed that in the Netherlands, per kilogram of standardized milk, the organic dairy cattle production system had a lower energy use and eutrophication potential than the conventional system, whereas the conventional system had a lower land use. Acidification potential and global warming potential were similar for both systems.

To achieve meaningful sustainable development, environmental impact assessment (EIA) should be performed to avoid the net losses in the environment resource base (55). The effects of agricultural systems on biodiversity are described by Aiama et al. (56). In industry (such as forestry and infrastructure), it is increasingly recognized that management of the operational and reputational risks due to water scarcity, pollution, climate change, and biodiversity loss is necessary. Therefore, compensation measures are necessary to restore the ecological balance or reduce the impact of industry. The authors also identify three main conditions under which biodiversity is so much threatened that it cannot be compensated:

(1) Agricultural systems with a large-scale impact on ecosystems and/or species in natural areas where regional biodiversity loss is occurring.
(2) When biodiversity protection measures for natural habitat areas and/or species are not properly designed or are not enforced effectively.
(3) When relevant biodiversity goals have no foundation in existing policies.

Unfortunately, boosting dairy production through scale enlargement and Holsteinization will generally lead to these three conditions. In India and Africa, the first goal of government is to enhance milk production (57) and environmental impact is of secondary importance. When compared to forestry and infrastructure, the agriculture industry has a far larger negative impact on biodiversity and the environment (56), and there is a need for more sustainable dairy production.

### Enhanced Use of Pesticides

During the exchange project, the negative effect of dairy development programs on biodiversity became clear in the so-called “cattle belt” of Uganda. Here, pastoralists live in small communities. To boost milk production, the local Ankole cow has increasingly been crossbred with Holstein Frisians. Generally, these crossbred cattle graze outside, and although they get relatively high-quality feed they also require intensive tick protection to survive. If not, the animals will suffer from tick-borne diseases, such as East Coast fever. The problem of tick-borne diseases is a threat to animal health, leading to large losses of (mainly exotic) livestock. The present solution to this problem is the two-weekly use of acaricide sprays on the cattle before they are sent into the field. This has led to spilling of pesticides into the environment with a negative effect on the biodiversity of insects and birds. Moreover, soil life is also negatively affected, as is the health of those exposed to these pesticides (58). Both acaricides and antibiotics are used without any form of quality control or restriction.

### Changes in Pasture Management

The extent to which biodiversity and the quality of the pasture can be threatened depends on the nature of the production system. In the Netherlands, the focus on high milk production (in kilogram of milk per year or lactation) has led to the scale enlargement of dairy farms, a sharp reduction in the number of farms, as well as to a huge impact on the environment. The pastures contain mostly highly productive grasses, such as English Ray grass, and a growing number of cows are kept indoors all the year round. Artificial fertilizers are used to produce high amounts of grass and silage for the cows, as well as manure mixed with urine that is injected into the soil (59). Manure injection has a negative impact on the soil life, while excess nitrogen and phosphorus from the manure has caused pollution of ground water and soil (60).

Monocultures of highly productive grasses have a negative impact on both plant and animal biodiversity. Since these grasses grow fast, early mowing is possible, which makes farmland birds more vulnerable for predators and removes the food on which chicks that feed on insects depend. In the Netherlands, the number of farmland birds in dairy farm areas has declined sharply over recent years (61).

### Loss of Local Cattle Breeds and Genetic Diversity

The state programs of India, Uganda, and Ethiopia (23, 26, 28) for boosting dairy production through crossbreeding with HFs contributes directly and indirectly to the increased use of antibiotics. Although this is unavoidable, efforts need to be geared toward reducing the use of antibiotics through the implementation of good management practices.

In most dairy development programs in developing countries, continued crossbreeding with HFs is one of the main ways of improving milk production. But crossbreeding leads to the loss of indigenous breeds and, moreover, to the loss of their special genetic traits that enable them to adapt to local environments, including resistance to indigenous disease and the ability to thrive on low-quality feed and lack of water (62). Crossbreds and imported pregnant heifers show high mortality due to disease. In Uganda, we spoke to a farmer who lost 100 of his 130 crossbreds in just 1 year. This leads not only to increased costs, as farmers have to buy replacements, but also to more poverty among farmers. Crossbreeding has been implemented in three of the four countries in the E-motive exchange project or it is being promoted throughout the country regardless of the different resources available and variety of agro-ecological zones. Meanwhile, the loss of genetic diversity within the HF breed and the negative effects of inbreeding on health and resilience is also a growing matter of concern (63).

Crossbreeding strategies require a proper evaluation of the local economic, social, and cultural conditions, and the role of farm animals in the community. Also, there is a need to develop a typology of production systems. A positive trend here is provided by an example from Ethiopia where strategic crossbreeding and breeding local breeds is considered as a two pronged approach to boosting production. Such an approach can counteract a lack of awareness about the conservation of farm animal genetic resources.

## Proposed Solutions

**Figure 2:**
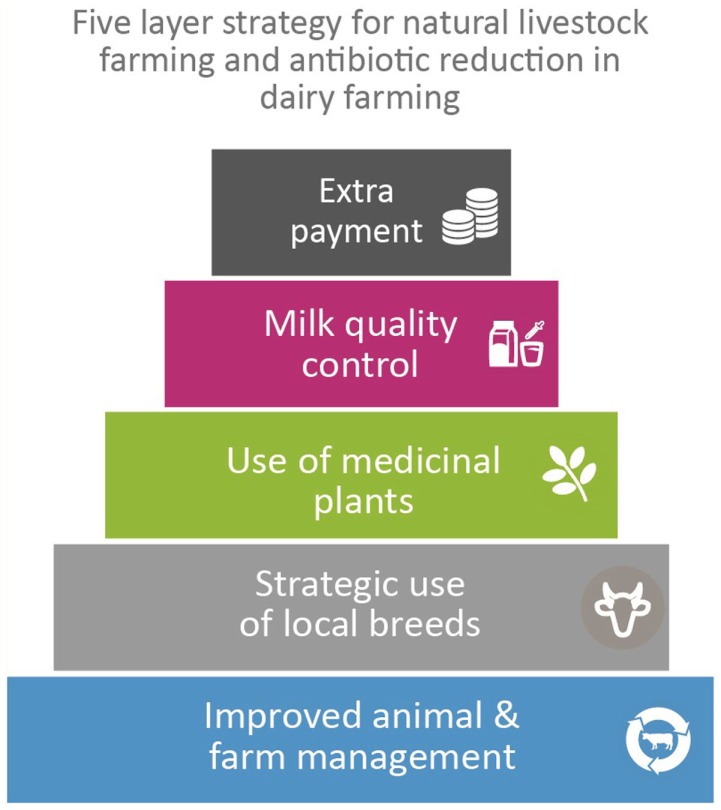
**The Natural Livestock Farming five-layered approach to reduce the use of antibiotics and other chemicals in dairy farming**.

There are diverse solutions to the complex problem of the excessive use of antibiotics and other agro-chemicals, such as acaricides, and these depend on the local situations. Meanwhile, on the basis of the experiences gained during the exchange project, we suggest a basic strategy consisting of a five-layered approach to reduce the use of antibiotics and other chemicals in dairy farming (Figure 2) (64) that includes the following elements.

### Awareness and Policy

- Increase awareness among researchers, policymakers, and NGO’s about the need to improve milk *quantity* together with milk *quality*. Raise consciousness about the link between dairy production methods and milk quality. Provide information on the current levels of residues from antibiotics and other agro-chemicals in dairy products, as well as their risk on human and environmental health.
- Reconsider or establish policies related to the free sale of antibiotics and other agro-chemicals, establish a rigorous control system for residues in products of animal origin.
- Facilitate dairy chains that include milk quality control on residues, and pay farmers a better price for residue-free milk.

### Improved Animal and Farm Management

- Boost dairy production mainly through improved animal management, including improved feeding, water, housing practices, and parasite control.
- Strengthen farm management environmental health by effective manure management, soil fertility and closing mineral (Nitrogen and Phosphorus) cycles.

### Ethno Veterinary Medicine

- Revitalize traditional knowledge on herbal medicine, train veterinarians and farmers, and develop scientific substantiation on remedies and practices concerning herbal medicine in dairy farming, gradually replacing antibiotics and other agro-chemicals with herbal products.

### Strategic Use of Local Breeds

- Genetic improvement of the local (dual purpose) cattle breeds with respect to milk production through selection, rather than crossbreeding them with exotic breeds such as HF. Though this process takes longer, it is more sustainable. Controlled crossbreeding under selected conditions, using innovative breeding strategies, such as three-way crosses.
- When continued crossbreeding has led to animals ill-adapted to local conditions, crossbreed the cows back with a local cattle breed. This can increase their adaptation to the climate and environment.

### Dairy Chain

- Establish milk quality control systems for residues in milk that are adapted to the existing (smallholder or pastoral) dairy system.
- Establish preference pricing system for residue-free milk.

## Conclusion

This article describes the hidden effects of dairy farming on public and environmental health from the perspective of the use of antibiotics and other agro-chemicals by comparing the situation in the Netherlands, India, Ethiopia, and Uganda. The threat of multi-resistant microbes and antimicrobial resistant (AMR) infections is still largely unaddressed within the dairy sector in India, Ethiopia, and Uganda, while the use of antibiotics and other agro-chemicals is rampant and largely uncontrolled. Therefore, raising awareness about the problem of antibiotic resistance, including the link with livestock production and the effect of the overall availability of agro-chemicals and their impact on environmental, animal, and human health is of utmost importance. Despite the differences in milk production, environmental problems occur in all four countries, while public health is threatened especially in countries with inadequate food (milk and meat) quality controls. Therefore, quality control programs for milk are needed to get healthy residue-free dairy products for these countries. It can also be concluded that dairy farming policies that only focus on the highest productivity per animal per year can lead to decreased animal health, increased use of agro-chemicals, negative impacts on the environment, and negative effects on milk quality and, consequently, human health. Revitalizing ethno veterinary medicine and the use of medicinal plants can be one of the ways to reduce antibiotic use, and reduce the use of agro-chemicals, as experiences in India show. Another important finding was that – for healthy dairy production – animals should be genetically well-adapted to their environment. This means that, in addition to strategic crossbreeding, a suitable breeding program for local breeds should also be implemented, together with improved animal- and farm management, including manure management and closed mineral cycles. The following step in this joint effort will be to integrate strict milk quality controls and extra payment for residue-free milk to farmers, thus, implementing the Natural Livestock Farming five-layer approach to reduce antibiotic use in dairy farming and to improve milk quality in the four countries.

## Author Contributions

All authors listed, have made substantial, direct and intellectual contribution to the work, and approved it for publication.

## Conflict of Interest Statement

The authors declare that the research was conducted in the absence of any commercial or financial relationships that could be construed as a potential conflict of interest.
