# Supplementary material for: The Hidden Effects of Dairy Farming on Public and Environmental Health in the Netherlands, India, Ethiopia, and Uganda, Considering the Use of Antibiotics and Other Agro-chemicals
Source: Front Public Health. 2016 Feb 24;4:12. doi: 10.3389/fpubh.2016.00012 (PMC4764701; doi:10.3389/fpubh.2016.00012)
Supplement: Supplementary file 1 [file Table_1.docx]

|  | **Netherlands** | **India** | **Ethiopia** | **Uganda** |
| --- | --- | --- | --- | --- |
| **Number of cattle** **Annual production** **Dairy farming system** | 1,6 million dairy cows  8,210 kg milk /year  between 20-40 litres/day  Specialized dairy, average 85 cows/farm | Cattle population 199 million, 62.4 million dairy cows, 105 million buffaloes.  Local cattle breeds (77 %) 2,1 kg/day  Crossbreeds 6-8 kg/day  Majority of smallholder integrated farms, with 2-3 dairy cows | Cattle population 52 million, 10.5 million dairy cows  305 liters/year  Pastoralist system: lowland grazing meat/milk system based on local breed; mixed crop-livestock systems heavily dependent on grazing(small holder grazing with local cattle and cross breds); intensive dairy farming in higher regions | Cattle population 14.4 Million 1.5 million dairy cows  2 billion liters of milk/ year. with indigenous cattle contributing 75% of the production. average for Ankole is 3litres/day. Crossbreeds give 5-7 liters per day.  Pastoralist in the cattle corridor, communal grazing in Northern and Eastern Uganda, paddocking in the Western Uganda and zero grazing in the peri-urban areas. |
| **Cattle Health problems** | Infectious endemic diseases controlled  Mastitis  Infertility  Lameness  Metabolic disorders  Calf scour | Infectious endemic diseases (e.g. FMD)  Mastitis  Bloat  Calf scour  Infertility  FMD  Enteritis  Post-partum complications (especially cross breeds)  Udder pox  Maggot wounds | Infectious endemic viral and tick-borne diseases (e.g. FMD, CBPP)  Pneumonia  Mastitis  Lameness  Infertility | Infectious endemic viral and tick borne diseases (e.g. East Coast fever)  Mastitis  Infertility  FMD  CBPP  LUMPY SKIN DISEASE  Metabolic diseases  Hardware disease in zero grazing |
| **Data on antibiotic use and resistance** | Farmers keep record of their antibiotic use  LEI Wageningen UR monitors the antibiotic use per animal species  FIDIN, the federation of the Dutch veterinary pharmaceutical industry, annually reports the overall sales of antibiotics  Animal Drug Authority collects data  Maran reports on use of antibiotics and resistance  Establishment of Animal Daily Doses (ADD) per year | Govt of India Food Safety & Standard Act (2006) prohibits residues in food – lack of implementation | Very limited information available; limited data and systematic surveillance; limited laboratory capacity | AMR in humans recognized as problem  Limited awareness of problem of antibiotic use in cattle; limited data and systematic surveillance; limited laboratory capacity |
| **Measures to protect the environ-ment** | Rules on the maximum amount of manure/fertilizer to be applied  Requirements to minimize NH_3_-emmission | Biodiversity regulations | No data | National drug act that gives guidelines on the use of antibiotics. The document is in place but implementation is lacking  Dairy Development Authority periodically tests milk for adulteration and residues.  National Environmental Management Authority(NEMA) asses environmental impact assessment |
| **Methods to reduce the use of antibiotics** | National programs on udder health and cow resilience  Farm health management program  Restriction on the use of certain antibiotics  Stable books with info on herbal treatments  Organic farming | Training of veterinarians and farmers on use of ethno- veterinary medicine (to limited extent)  Promote organic dairy farming | None | None |
| **Environ-mental problems** | Lack of biodiversity in pasture  Reduction/disappearance of insects, birds, soil life  Lack of biodiversity in soil  Obligatory injection of manure in soil  MRSA and ESBL in surface water  Pollution of ground and surface water | High levels of residues in milk in the food chain, environment | Cow manure majorly used for cooking.  Overgrazing in open access communal pastures | Residues of acaricides and veterinary medicine in environment  Reduction/disappearance of insects, bees, butterflies, birds, soil life |
| **Use of herbal medicine** | Is increasing (48 % of farmers use herbal medicine) | Large databases on herbal medicine;  Training of veterinarians and farmers on use of ethno- veterinary medicine | Documentation work done for specific pastoral region. More documentation and field verification needed; Ethiopian drug policy failed to address ethno veterinary practices and medicines | Pastoral knowledge on herbal medicine is declining |
| **Breeding** | Mainly HF, some local breeds  Mostly HF since 1960’s, trend to crossbreeds back with local or European dual purpose breeds to increase robustness | Mainly HF to pure breed crossbreed cattle  Loss of 50% of local breeds  Systematic crossbreeding mainly with HF and some Jersey since 1964 | Total 32 local breeds, HF crossbreeds  Crossbreeding with HF and Jersey since 1990’s  Community based breeding program initiated and in progress | 93.6% of cattle local breeds (e.g. Ankole),  Crossbreeding with HF, Jersey, Guernsey and Ayrshire is increasing |
| **Milk control** | Strictly regulated by Qlip  At farm-level 0.016% of the tested samples positive on antibiotic residues | Control on residues of veterinary drugs at level of milk factory is lacking at community milk collection centers  Lack of enforcement | No stringent regulation structure in place; Lack of control on residues of veterinary drugs | Regulated by Dairy Development Authority(DDA),Very low enforcement. Building capacity for milk testing at every milk collection center |
| **Develop-ments** | Antibiotic reduction strategy developed between ministries of health, agriculture and environment | National Dairy Development Plan  Intensive Dairy Development Program,  Strengthening Infrastructure for Quality and Clean Milk Production,  Assistance to Cooperatives  Dairy Entrepreneurship development Scheme. | Dairy development programs  Growth and Transformation Plan | Dairy industry Act and Stature for Dairy is guiding dairy development  Dairy development authority |
| **Needs** | Need to develop suitable methods of enforcement of antibiotic reduction  Knowledge of preventive animal health care and alternative (herbal) medicine  Research and substantiation of the working of herbal medicine  New breeding goals aiming at improving cow health, welfare, fertility and longevity  Development of a premium on milk that is produced without antibiotics | Need to develop suitable methods of enforcement of antibiotic reduction and controlled antibiotic sales and use by unauthorized practitioners  Milk control on residues at community dairy collection centre  Develop programme for awareness and training for farmers and the other stakeholders | Include herbal medicines as a means to reduce synthetic antibiotic use.  Improve productivity of local breeds (feeding, selection)  Test systems for antibiotics in milk  Evaluate local indigenous knowledge in ethno veterinary medicine to develop herbal medicines for dairy cattle  Breeding programs for local breeds that are more resistant to diseases | Herd Nutrition and water for production  Improvement in Animal Breeds and genetics  Analysis of drug potency, use and residues in animal products  Herbal Medicine revitalization  Capacity Building for Scientists in adaptive research and innovations  Establishment of field milk testing sites  Building capacity of stakeholders along the milk values chain in proper milk handling and quality control.  Development of quality based payment systems of milk. |
